# Case report: Effectiveness of brexpiprazole and esketamine/ketamine combination: A novel therapeutic strategy in five cases of treatment-resistant depression

**DOI:** 10.3389/fpsyt.2022.890099

**Published:** 2022-07-22

**Authors:** Lai Fong Chan, Luke Sy-Cherng Woon, Nuur Asyikin Mohd Shukor, Choon Leng Eu, Nurazah Ismail, Song Jie Chin, Nik Ruzyanei Nik Jaafar, Azlin Baharudin

**Affiliations:** ^1^Department of Psychiatry, Faculty of Medicine, National University of Malaysia, Kuala Lumpur, Malaysia; ^2^Department of Psychiatry, Hospital Canselor Tuanku Muhriz, National University of Malaysia, Kuala Lumpur, Malaysia; ^3^Psychiatry Unit, Department of Medicine, Faculty of Medicine and Health Sciences, Universiti Sains Islam Malaysia, Nilai, Malaysia

**Keywords:** treatment-resistant depression, effectiveness, ketamine, brexpiprazole, esketamine

## Abstract

A significant proportion of patients with treatment-resistant depression do not attain functional recovery despite administration of multiple steps of pharmacotherapeutic strategies. This highlights the elusiveness of meeting unmet needs in existing pharmacotherapies for treatment-resistant depression. There is accumulating evidence that antidepressant agents involving the glutamatergic system such as brexpiprazole and esketamine/ketamine have more rapid onset of action and potentially improved effectiveness as an augmentation therapy in treatment-resistant depression. This case series aimed to report five complex cases of unipolar and bipolar treatment-resistant depression where conventional treatment strategies were inadequate in managing high risk suicidal behavior and achieving functional recovery. We discussed further the possible synergistic mechanisms of the novel combination strategy of brexpiprazole and esketamine/ketamine, clinical and patient factors that influenced treatment response, challenges with this combination strategy and implications for future practice and research.

## Introduction

Unipolar treatment-resistant depression (TRD) is most commonly defined as non-response to at least two adequate courses of sequential antidepressant treatment in a single major depressive episode (1). Bipolar TRD is considered more treatment-refractory and has been defined as failure of sustained symptomatic remission for 8 consecutive weeks after 2 different treatment trials of adequate therapeutic doses, with at least two recommended monotherapy treatments; or at least 1 monotherapy treatment and another combination treatment (2). There is an urgent need for improving the evidence base with regards to our current limited pharmacological options for unipolar and bipolar TRD in view of this population's significant risk of morbidity and mortality, including suicidal behavior (3).

Ketamine, an anesthetic drug; and brexpiprazole, an atypical antipsychotic, are novel pharmacological agents with potential advantages in improving cognition and functionality in treatment-resistant depression. A systematic review by the Canadian Network for Mood and Anxiety Treatments (CANMAT) Task Force found Level 1 evidence for single dose intravenous ketamine as a third-line agent for unipolar TRD. The evidence for multiple IV ketamine doses in unipolar TRD is limited to Level 3 (4). The U.S. Food and Drug Administration (FDA) has approved esketamine, the S-enantiomer of ketamine, as an add-on intranasal therapy to an oral antidepressant in treatment-resistant unipolar depression patients and major depressive disorder (MDD) with suicidal ideation or behavior (5, 6). In terms of treatment-resistant bipolar depression, a handful of studies supported the efficacy of single infusion ketamine, although the meta-analytic evidence for longer term ketamine treatment is mixed (7). Brexpiprazole is FDA-approved for adjunctive treatment of major depressive disorder, with a number needed to treat (NNT) of 12 and a number needed to harm (NNH) of 53 according to a systematic review by (6, 8). Currently, there is a paucity of real-world data on the effectiveness of brexpiprazole in treatment-resistant unipolar and bipolar depression beyond clinical trials (9). To the best of our knowledge, there are no published pre-clinical or clinical studies of brexpiprazole and ketamine/esketamine combination for the treatment of depression. The rationale of this combination is aligned with the Window of Antidepressant Response Paradigm (WARP) (10). Malhi et al. proposed the WARP concept whereby esketamine, as a glutamatergic rapid-acting antidepressant, could potentially be utilized within the immediate-response window in combination with brexpiprazole, a serotonin-dopamine modulator with partial D_2_ receptor agonism within the fast-response window as an adjunctive strategy to overcome the delay in conventional selective serotonin reuptake inhibitor (SSRI) or serotonin and norepinephrine reuptake inhibitors (SNRI) antidepressant response (10).

The objective of our case series is to describe the efficacy, tolerability and functional outcome of a novel pharmacological approach involving brexpiprazole as adjunctive treatment to either maintenance intravenous ketamine or intranasal esketamine; in addition to standard biological and psychosocial interventions, in three unipolar and two bipolar TRD patients at an urban public university hospital in Malaysia, an upper middle-income country in South-East-Asia.

## Case report 1

**Figure 1:**
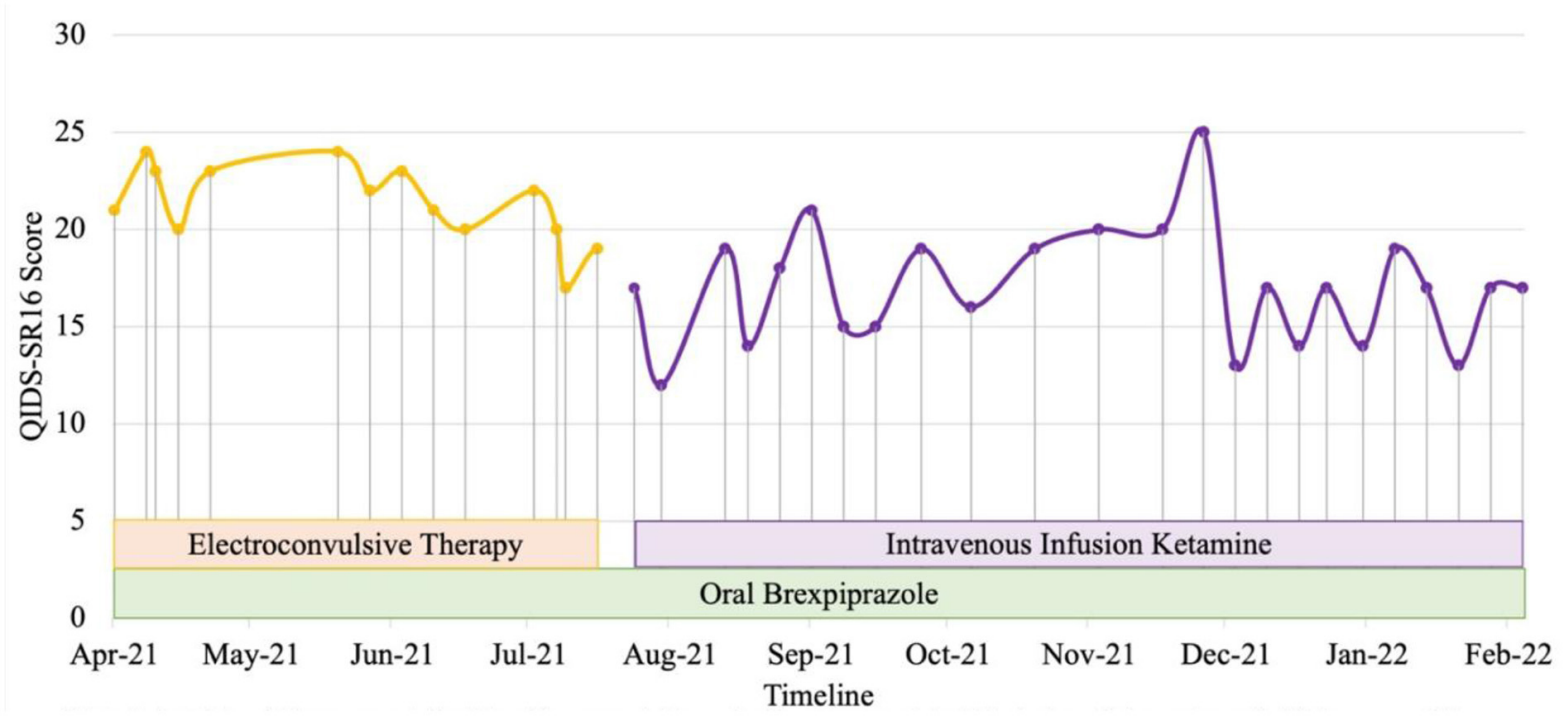
Case 1 timeline for longitudinal course of treatment response. Patient 1 is a Malay woman in her 20s with a current diagnosis of treatment-resistant bipolar type II depression with lifetime co-morbid diagnoses of complex post-traumatic stress disorder, obsessive-compulsive disorder and borderline personality disorder. Brexpiprazole was initiated at the dose of 0.5 mg and titrated to a maximum of 4mg. Intravenous infusion ketamine 0.5 mg/kg is initiated at July 2021 to replace ECT due to cognitive impairment as a side effect of ECT. Co-medications include lamotrigine 200 mg, sodium valproate 2 g, quetiapine 300 mg, and clonazepam 2 mg PRN. Currently, she has lesser obsessive, PTSD and depressive symptoms, including marked reduction in intensity and frequency of suicidal thoughts with improvement in her functionality.

Patient 1 is a Malay woman in her 20's. Previous diagnoses included major depressive disorder, complex post-traumatic stress disorder (PTSD), obsessive-compulsive disorder (OCD) and borderline personality disorder (BPD). She experienced unremitting depression with chronic thoughts of self-harm and suicide since 2017. She had been unemployed since she was unable to complete her health-care professional internship in 2020. Systematic physical examination revealed no significant finding. Comprehensive review to rule out potential factors of pseudo treatment resistance depression including presence of substance use disorder and treatment non-adherence were performed. Previous trials of selective serotonin reuptake inhibitors (SSRI) intensified her suicidal ideation and aripiprazole augmentation were unsuccessful. She experienced a severe relapse of a major depressive episode in 2020 with multiple self-harm attempts precipitated by triggers that evoked memories of previous childhood trauma with commanding auditory hallucinations to end her life. Electroconvulsive therapy (ECT) was initiated in view of the high risk of suicide. Her diagnosis was revised to treatment-resistant bipolar type II depression when hypomanic episodes with irritability, reduced need of sleep, increased energy, and increased goal directed activities emerged in December 2020. Trauma-focused psychotherapy was also commenced. She was hospitalized for a suicide attempt during a subsequent severe depressive relapse in March 2021. Brexpiprazole was added to sodium valproate and lamotrigine to target severe depressive symptoms with mood congruent psychosis in the context of bipolar disorder in addition to maintenance ECT. Her mood and obsessive symptoms reduced, but she experienced distressing cognitive impairment on maintenance ECT. In July 2021, ECT was stopped, and her treatment regime was optimized to include intravenous ketamine (0.5 mg/kg, 90-min infusion, weekly to 2-weekly), brexpiprazole 4 mg ON, sodium valproate prolonged release tablet 2 g ON, lamotrigine 200 mg ON, quetiapine 300 mg ON and clonazepam 2 mg PRN, in combination with psychotherapy. Her depression level reduced from very severe to moderate (Self-report Quick Inventory of Depressive Symptomatology, QIDS SR16 scores of 22 to 13) within 5 months (Figure 1). Currently, her mood has significantly stabilized with less debilitating obsessive and PTSD symptoms, as well as a marked reduction in the intensity and frequency of suicidal thoughts. She is also pursuing her interest in baking and realizing her entrepreneurial potential with supported employment.

## Case report 2

**Figure 2:**
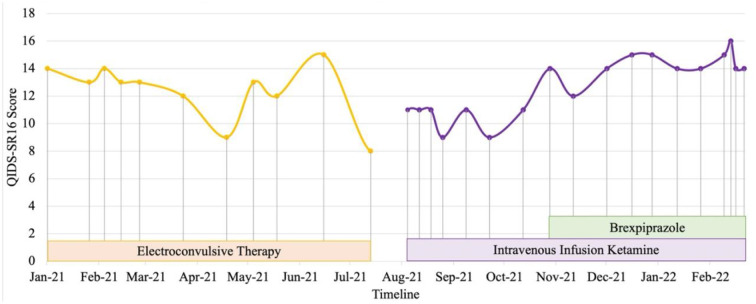
Case 2 timeline for longitudinal course of treatment response. Patient 2 is a male in his 20's with a current diagnosis of treatment-resistant bipolar type II depression. Brexpiprazole was initiated at the dose of 0.5 mg and titrated to a maximum of 3 mg in combination with intravenous infusion ketamine 0.5 mg/kg. Co-medications include lamotrigine 300 mg, quetiapine 800mg, vortioxetine 20 mg and clonazepam 0.5 mg with cognitive behavioral therapy and family perticipation in online family support group. Currently, despite fluctuations in mood, he expressed improved levels of satisfaction and self-efficacy in terms of occupational functioning.

Patient 2 is a health-science graduate in his 20s. He experienced recurrent brief hypomanic symptoms during his undergraduate studies such as feeling energetic, having reduced need of sleep, and spending spree which lasted for about 2 days. He experienced a severe major depression (persistent low mood, anhedonia, hopelessness, worthlessness, social withdrawal and suicidal thoughts) since 2018 and was unable to proceed with internship upon graduation. He was referred to our center in 2020 for inpatient care after an aborted suicide attempt with a lethal method. Systematic physical examination revealed no significant finding. He was diagnosed with bipolar type II depression during the admission. Previous treatment with escitalopram was unsuccessful. Vortioxetine, quetiapine and lamotrigine were titrated to optimize mood stability as monotherapy with either agent was inadequate to control depressive and hypomanic symptoms. Comprehensive review to rule out potential factors of pseudo treatment resistance depression including presence of substance use disorder, personality disorders and treatment non-adherence were performed. After a serious suicide attempt 4 months later, ECT was commenced. His mood stabilized and suicidal behavior reduced with the addition of maintenance ECT and cognitive behavior therapy (CBT). After 9 months of the above treatment, he was concerned about the impact of ECT cognitive side-effects on his occupational functioning and future career prospects. In view of the significant risk of relapse and suicide, he gave informed consent to switch from maintenance ECT to off-label intravenous ketamine. His depression severity reduced from moderate to mild (QIDS SR16 scores from 11 to 9) after titration of intravenous ketamine (0.5 mg/kg, 90-min infusion) to weekly doses for 6 weeks (Figure 2). He experienced a depressive relapse precipitated by job insecurity and high-expressed emotion in his family. Despite fluctuations in mood, he expressed improved levels of satisfaction and self-efficacy in terms of occupational functioning after adding brexpiprazole 0.5 mg ON and subsequently titrated to maximum of 3 mg ON to vortioxetine 20 mg ON, quetiapine 800 mg ON, lamotrigine 300 mg ON, clonazepam 0.5 mg PRN, and intravenous ketamine twice a month. CBT is ongoing and his family has started participating in an online family support group.

## Case report 3

**Figure 3:**
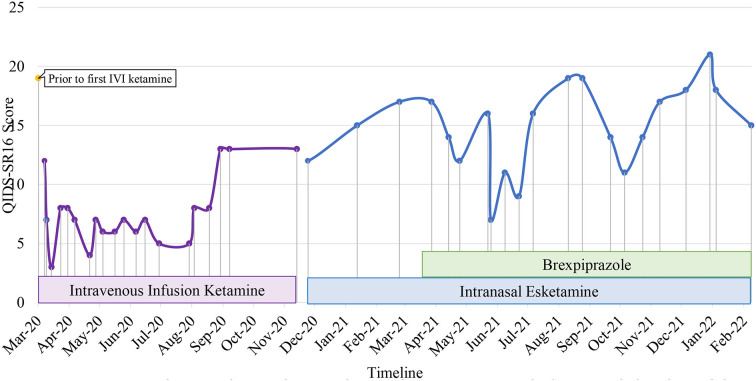
Case 3 timeline for longitudinal course of treatment response. Patient 3 is a single woman in her 30s' with a current diagnosis of treatment-resistant unipolar depression with physical co-morbidity of bilateral normal tension glaucoma with high myopia. Brexpiprazole was initiated at the dose of 0.5 mg and titrated to a maximum of 3.5 mg in combine with intranasal esketamine 84mg fortnightly. Co-medications include zolpidem 10 mg, mirtazapine 45 mg, quetiapine 800 mg, vortioxetine 20 mg, and clonazepam 6 mg. There was an overall reduction of depressive symptoms after initiation of ketamine, albeit with fluctuations of these symptoms, perpetuated by psychosocial stressors over the longitudinal course of treatment. She reported more sustainable improvement in her energy, personal care, interpersonal and occupational functioning after brexpiprazole was added to address this issue.

Patient 3 is a single healthcare worker in her 30's with a diagnosis of TRD. She presented to our center in May 2019 with worsening depressive symptoms for 4 months and intense suicidal ideation perpetuated by increased interpersonal conflicts. She had low mood, poor sleep, hopelessness, worthlessness, loss of energy and appetite, and difficulty concentrating for 4 years. She experienced multiple stressors including a burglary, workplace and family interpersonal problems, and bilateral normal tension glaucoma undergone bilateral trabeculectomy and high myopia. There were no manic, hypomanic, psychotic symptoms nor history of substance abuse. Systematic physical examination revealed no significant finding. Since 2016, her significant depressive symptoms persisted despite adequate trials of agomelatine, mirtazapine, fluvoxamine, vortioxetine, aripiprazole, quetiapine, in addition to a brief psychodynamic psychotherapy and hypnotherapy. Comprehensive review to rule out potential factors of pseudo treatment resistance depression including presence of substance use disorder, personality disorders and treatment non-adherence were performed. In 2019, she received acute and maintenance ECT in our inpatient center which significantly reduced her suicidal behavior i.e., resolution of suicidal plans and attempts. However, her depression did not fully remit, compounded by occupational challenges and interpersonal relationship issues. Hence, she was started on intravenous ketamine (0.5 mg/kg, 90-min infusion) in March 2020. The most notable transient side effects during the procedure were dizziness, dry mouth, and dry eyes. She was then switched to maintenance esketamine 84 mg fortnightly when it became available in December 2020. She experienced new side effects, i.e., mild burning sensation in the throat and double vision occurring about 10 min post nasal esketamine administration, which resolved spontaneously within 50 min of observation. However, the effect was short-lived as the negative cognition reemerged by the end of 2nd week post-esketamine administration. There was an overall reduction of depressive symptoms, including suicidal and ruminating thoughts after initiation of ketamine, albeit with fluctuations of these symptoms, perpetuated by psychosocial stressors over the longitudinal course of treatment (Figure 3). Brexpiprazole was subsequently added to address this issue and titrated from 0.5 mg to 3.5 mg, after which she reported more sustainable improvement in her energy, personal care, interpersonal and occupational functioning whereby she was able to establish and sustain her own business.

## Case report 4

**Figure 4:**
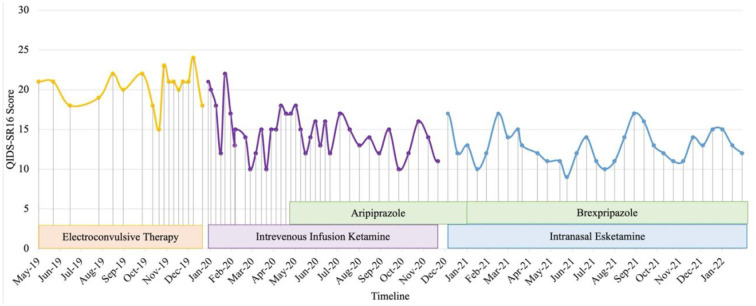
Case 4 timeline for longitudinal course of treatment response. Patient 4 is a woman in her 40's with a current diagnosis of treatment-resistant unipolar depression with physical co-morbidity of congenital glaucoma and bilateral complete vision loss. Brexpiprazole was initiated at the dose of 0.5 mg and titrated to a maximum of 1mg in combine with intranasal esketamine 84 mg fortnightly. Co-medications include mirtazapine 30 mg, agomelatine 50 mg. Her mood stability and functionality improved with the combination of brexpiprazole and esketamine in her pharmacological regime and Acceptance and Commitment Therapy (ACT)-based psychotherapy.

Patient 4 is in her mid-forties with congenital glaucoma and complete vision loss since she was 6 years old. She developed significant depressive symptoms in her mid-thirties with persistent low mood, anhedonia, poor concentration and excessive guilt. Premorbidly, she was highly independent and functioning very well-occupationally. She sought treatment at our center in February 2019 when she experienced frequent and intense suicidal ideation. Systematic physical examination revealed no significant finding. Previous adequate trials of mirtazapine, agomelatine and vortioxetine were not adequately effective. Comprehensive review to rule out potential factors of pseudo treatment resistance depression including presence of substance use disorder, personality disorders and treatment non-adherence were performed. She received 15 bilateral ECT sessions, which were ineffective and caused significant cognitive impairment. Therefore, intravenous ketamine (0.5 mg/kg, 90-min infusion) was initiated in January 2020 which significantly reduced suicidal ideation intensity. Intravenous ketamine was switched to esketamine in December 2020 when the latter was available. Esketamine was initiated at 56 mg 2-weekly which continued to reduce her suicidal thoughts. She experienced tolerable side-effects such as transient dissociation and headache. Her treatment regime also included mirtazapine 30 mg and agomelatine 50 mg. In addition, aripiprazole 2.5 mg was switched to brexpiprazole 1 mg OD in March 2021 due to intolerable side effects such as lethargy and akathisia. Brexpiprazole improved her anxiety symptoms though it could not be further optimized due to lethargy at higher doses. Esketamine was further optimized to 84 mg every 2-weekly in June 2021. Her mood stability and functionality improved with the combination of brexpiprazole and esketamine in her pharmacological regime and Acceptance and Commitment Therapy (ACT)-based psychotherapy (Figure 4).

## Case report 5

**Figure 5:**
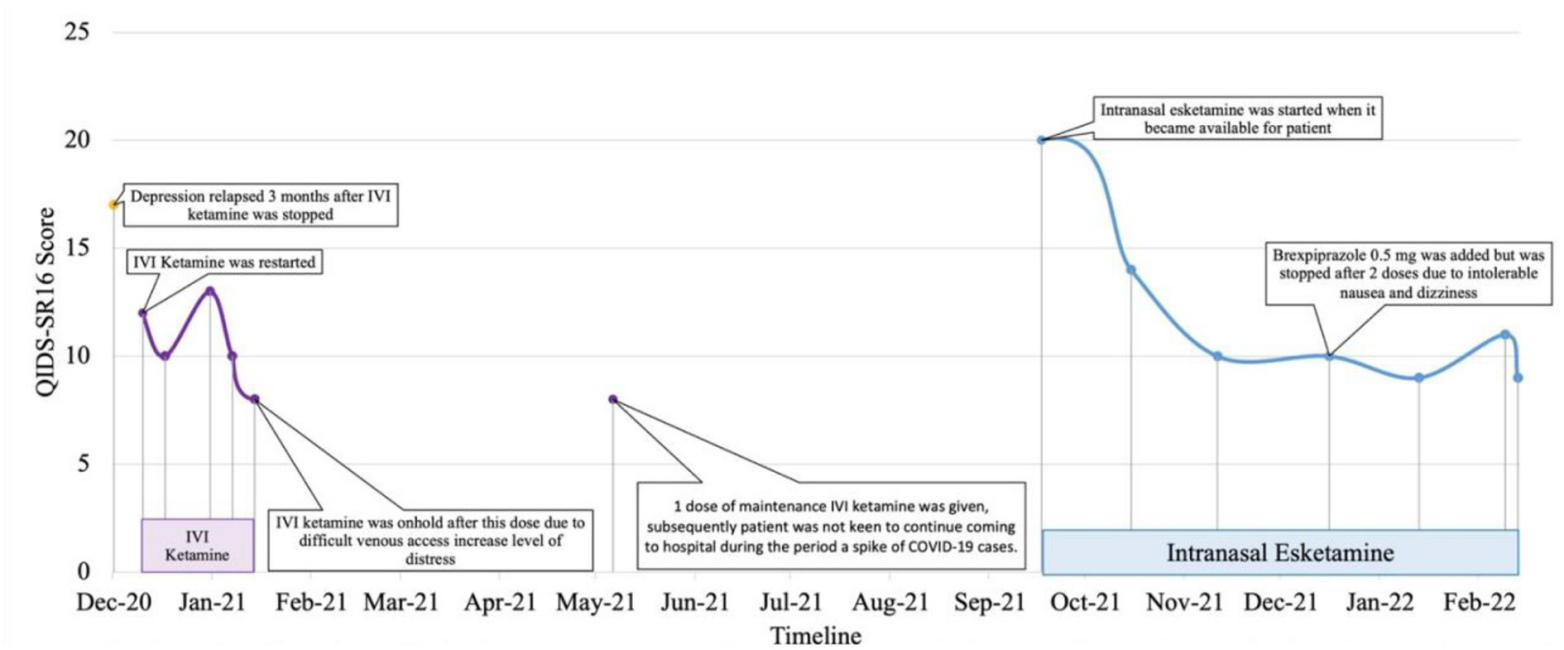
Case 5 timeline for longitudinal course of treatment response. Patient 5 is a Chinese female in her late 50's with a current diagnosis of treatment-resistant unipolar depression with physical co-morbidity of sacral fracture. She was on IVI ketamine since 2012 with symptomatic remission as reported in another published case series. IVI ketamine was witheld on July 2020 in view of patient's concern of contacting with COVID-19 cases during hospital visit. Her depression relapsed on Dec 2020 where IVI ketamine was restarted and subsequently reduction of QIDS-SR16 score. Co-medications include esketamine 56mg fortnightly, agomelatine 50 mg, quetiapine 800 mg, mirtazapine 45 mg, lorazepam 1 mg BD, zolpidem 10 mg, and clonazepam 2 mg PRN. Her depressive symptoms and functioning improved after 3 months on intranasal esketamine.

Patient 5 is a Malaysian Chinese retired educator in her late 50's with a 30-year history of major depressive disorder with anxious distress and recurrent suicidal ideation. Systematic physical examination revealed no significant finding. Comprehensive review to rule out potential factors of pseudo treatment resistance depression including presence of substance use disorder, personality disorders and treatment non-adherence were performed. She was diagnosed with treatment-resistant depression since 2001. Cognitive behavior therapy, family psychoeducation and a total of 131 ECT administrations (79 bilateral and 52 unilateral) were given from 2001 to 2012. Her depressive symptoms partially remitted but she experienced residual cognitive deficits post-ECT which severely impacted her functioning. A previous case series documented her symptomatic remission and significant functional improvement of TRD on intravenous ketamine treatment from 2012-2018 with minimal and transient side-effects (sedation, nausea and dizziness) that resolved within a week (11). Monthly outpatient maintenance intravenous ketamine was withheld in July 2020 due to concerns about contracting COVID-19 from monthly hospital visits. Her depression relapsed (QIDS SR16 score = 12, moderate severity) precipitated by disability due to a sacral fracture and high-expressed emotion in her family. After resuming monthly maintenance ketamine in December to February 2021, she achieved remission (QIDS SR16 score = 8). Maintenance intravenous ketamine was temporarily put on hold from February to May 2021 due to challenging intravenous access which increased her level of distress pre-ketamine infusion. After receiving one maintenance dose of intravenous ketamine in May 2021, the patient was not keen to continue coming to the hospital for treatment during the period of a spike of COVID-19 cases (12). In September 2021 (QIDS SR16 score = 20, severe depression), she was switched to intranasal esketamine 28 mg monthly based on her preferred frequency of hospital visits. Her depressive symptoms and functioning improved after 3 months (QIDS SR16 score = 9, mild depression) though dose optimisation had to be gradually done in view of transient dizziness post-esketamine. In December 2021, Brexpiprazole 0.5 mg on was added to target residual depressive symptoms such as insomnia but was stopped after two doses due to intolerable nausea and dizziness. Since February 2022, she has been tolerating esketamine 56 mg od well with no adverse events (QIDS SR_16_ score = 9, mild depression) in combination with agomelatine 50 mg ON, quetiapine extended release 800 mg ON, mirtazapine 45 mg ON, lorazepam 1 mg BD, zolpidem 10 mg ON, and clonazepam 2 mg PRN (Figure 5).

## Discussion

Based on our case series, the majority (two unipolar and twobipolar) of TRD patients demonstrated significant improvements in depressive symptoms including suicidal behavior, cognition and functionality with a combination of brexpiprazole and maintenance intravenous ketamine or intranasal esketamine as part of their treatment regime. One patient with unipolar TRD discontinued brexpiprazole due to intolerable dizziness. We employed an infusion regime of 0.5 mg/kg over a longer period of 90 min in our LMIC setting instead of the conventional 40 min as a pragmatic cost effective and less labor-intensive approach without the requirement of full anesthesia monitoring for serial ketamine infusions based on study by Rasmussen et al. (13). All five patients gave written informed consent for publication of the respective case reports.

While brexpiprazole is approved by the FDA as an augmenting agent for MDD patients with ‘inadequate response' to standard antidepressant treatments, the evidence-base for the most severe cases of bipolar TRD is currently lacking. An open-label pilot study of brexiprazole (4 mg maximum dose) for bipolar depression by Brown et al. showed improvements in depressive symptoms and quality of life (14). Evidence suggests that through N-methyl-D-aspartate blockade, esketamine upsurges glutamate release leading to increases in α-Amino-3-hydroxy-5-methyl-4-isoxazolepropionic acid (AMPA) receptors stimulation and a rise in neurotrophic signaling that restore synaptic function in brain areas involved with the regulation of mood and emotional behavior. Brexpiprazole, a partial dopamine agonist, also potentiates neurotransmission via the AMPA receptors. However, in contrast to esketamine/ketamine; brexpiprazole also acts on the monoamine pathway; with a faster antidepressant effect than SSRI alone. A single administration of the combination of fluoxetine and brexpiprazole significantly increased the D-serine/L-serine ratio in the frontal cortex, just as a single dose of racemic ketamine (15). Another noteworthy clinical observation of this combination is the apparent cognitive benefits that improve functionality. Pro-cognitive properties of ketamine and brexpiprazole as individual pharmacological agents have been demonstrated by previous preclinical and clinical studies. Cognitive deficits in mice induced by phencyclidine was mitigated by ketamine via activation of brain-derived neurotrophic factor-tropomycin receptor kinase B (BDNF-TrkB) signaling, as well as brexpiprazole via serotonin 1A (5-HT_1A_) receptors (16, 17). A review by Gill et al. found that subanaesthetic doses (0.5 mg/kg) of intravenous ketamine significantly improved working memory, learning memory, speed of processing and verbal learning memory in TRD patients (18). Improvements in general and cognitive functioning of patients with major depressive disorder treated with adjunctive brexpiprazole was shown in an open-label study by Fava et al. (19). In addition, there is increasing evidence of the efficacy of ketamine as a rapid-acting anti-suicidal agent. Patients at very high risk of suicide, particular with bipolar disorder, significantly achieved remission of suicidal ideation with acute intravenous ketamine (20). Currently, there is very limited published data on the role of brexpiprazole as an anti-suicidal medication (21). Future systematic studies are warranted to investigate the potential synergism of ketamine and brexpiprazole as acute and long-term interventions in preventing suicide.

The combination of esketamine/ketamine and brexpiprazole may pose some challenges in view of overlapping side effects. Common adverse events associated with esketamine/ketamine for TRD include vestibular effects (e.g., dizziness, nausea, and vomiting), sympathomimetic symptoms (e.g., tachycardia, hypertension), and psychomimetic symptoms (e.g., hallucinations, dissociative symptom) (Table 1) (22). Based on pharmacovigilance data from the the FDA Adverse Event Reporting System (FAERS), Gastaldon et al. (2021) have raised concerns over esketamine-related serious adverse events such as suicidal ideation and suicide deaths in which the former remained significant when compared to venlafaxine (23). These authors also reported that serious adverse events were more common in cases of esketamine co-medication with antipsychotics, mood stabilizers, benzodiazepines. Other authors have argued that the causal role of esketamine in increasing suicidal risk is still uncertain (24). Nevertheless, this phenomenon underscores the urgency of further research and continued vigilant monitoring of suicidal behavior in patients on esketamine, particularly when combined with other medications (25). Findings from systematic review and meta-analysis suggest that intravenous racemic ketamine was superior in terms of efficacy and treatment retention rates compared to esketamine for unipolar and bipolar treatment resistant depression (26). These authors highlighted the need for future research, including substantively more head-to-head trials of intravenous racemic ketamine vs. esketamine for unipolar and bipolar depression to further elucidate the underlying reasons and mechanisms of such differences in efficacy and acceptability. The evidence is still mixed on whether esketamine has a better tolerability profile compared to intravenous racemic ketamine, compounded by study heterogeneity which complicates arriving at definitive conclusions about the effectiveness of intravenous vs. intranasal ketamine (5, 26, 27). More recently, arketamine, another distinct ketamine enantiomer, has shown some initial potential in animal models and Leal et al's open-label pilot study as a rapid-onset intravenous antidepressant, possibly with a higher response and remission rate and more sustained effects with a better tolerability profile than intravenous racemic ketamine and intranasal esketamine (28). However, these findings need to be confirmed with larger systematic and controlled studies in the future. Postulated mechanisms of action from pre-clinical studies hypothesize that arketamine's antidepressant effects occur via the activation of α-amino-3-hydroxy-5-methyl-4- isoxazolepropionic acid (AMPA) receptors as well as subsequent activation of brain-derived neurotrophic factor (BDNF)- tropomyosin receptor kinase B (TrkB) signaling effect, independent of NMDA receptor antagonism as the basis of racemic ketamine and esketamine. According to a meta-analysis by Kishi et al., akathisia, somnolence and weight gain were significant side effects of brexpiprazole (29). In addition, dizziness is another potential side effect although its incidence was not significantly higher compared to placebo. However, adverse event susceptibility varies among individuals and the risk of multiple drug interaction exacerbating overlapping side effects should be considered. This phenomenon is illustrated in case report 5, whereby transient dizziness due to esketamine and ketamine seemed to be exacerbated to an intolerable level with the addition of brexpiprazole which had to be discontinued. Current expert consensus guidelines recommend vigilant surveillance of cognitive changes, vital signs, genitourinary toxicity, hepatic toxicity, progression of suicidal behavior, as well as the risk of abuse liability in patients on maintenance esketamine/ketamine treatment (5, 30). The anecdotal findings from our retrospective case series are limited by a small sample size. Future systematic controlled studies are required to established the effectiveness of the combination of brexpiprazole with ketamine/esketamine.

**Table 1:** The profile of ketamine/esketamine and brexpiprazole.

	**Ketamine**	**Esketamine**	**Brexpiprazole**
Molecular composition/ possible mechanism of action	A racemic mixture of two enantiomers, R-ketamine and S-ketamine/ NMDA receptor antagonist	A purified S-enantiomer/ NMDA receptor antagonist	Serotonin-dopamine activity modulator; dopamine D2 and serotonin 5-HT1A partial agonist; also potentiates neurotransmission via the AMPA-receptors
Route of administration	Intravenous infusion	Nasal, using metered dose spray	Oral
Clinical indications	An anesthetic that has been used off-label for treatment-resistant unipolar and bipolar depression and suicidal behavior	FDA-approved for treatment-resistant unipolar depression and major depressive disorder with suicidal ideation and behavior	FDA-approved for augmentation for inadequate response in treatment of depression
Adverse effects	Vestibular, sympathomimetic & psychotomimetic		Akathisia, somnolence, weight gain, dizziness

Sustainable access to esketamine/intravenous ketamine and brexpiprazole is another major challenge. Upscaling of maintenance intravenous ketamine treatment for TRD patients locally is hampered by the labor-intensiveness of service delivery as well as space limitations compounded by the need for adequate physical distancing during the COVID-19 pandemic. In addition, access to non-generic brexpiprazole and esketamine in our case series is based on industry-supported pro-bono compassionate grounds, thus calling into question long-term cost affordability. Future cost benefit analysis of brexpiprazole and esketamine/ketamine combination therapy within healthcare policies is crucial in mitigating the significant economic burden of TRD.

## Conclusion

This case-series has highlighted the effectiveness of combining brexpiprazole and maintenance esketamine/intravenous ketamine in the treatment regime for unipolar and bipolar patients with treatment-resistant depression. Effectiveness of this combination appears to be a promising, especially in terms of reducing suicidal behavior and improving cognition as well as functional recovery. Challenges include individual-level sensitivity to specific overlapping adverse events, sustainability in terms of service capacity and cost-effectiveness. Future pragmatic research is required to establish the real-world feasibility of including brexpiprazole and maintenance esketamine/ketamine combination in our therapeutic armamentarium for treatment-resistant depression.

## Data availability statement

The original contributions presented in the study are included in the article further inquiries can be directed to the corresponding author.

## Ethics statement

Written informed consent was obtained from the individual(s) and/or next of kin for the publication of this case report and any potentially identifiable data included in this article.

## Author contributions

NN and LC contributed to conceptualization. LC, LS-CW, NM, CE, NI, and NN wrote the first draft. LC, LS-CW, NM, CE, NI, SC, NN, and AB contributed to the intellectual content, revised, and reviewed the final draft. All authors contributed to the article and approved the submitted version.

## Funding

Brexpiprazole and esketamine were supplied pro-bono to the patients in these case series by Lundbeck and Johnson and Johnson, respectively via patient compassionate programs. The selection and clinical management of patients in this case series were done independently by the respective treating clinicians with no financial compensation from Lundbeck or Johnson and Johnson.

## Conflict of interest

Brexpiprazole and esketamine were supplied probono to the patients in these case series by Lundbeck and Johnson and Johnson, respectively via patient compassionate programs. The selection and clinical management of patients in this case series were done independently by the respective treating clinicians with no financial compensation from Lundbeck or Johnson & Johnson. Apart from this case series, LC, LS-CW, CE, NN, and AB have received conference sponsorships, speaker and/or consultancy honoraria from Lundbeck and/or Johnson & Johnson. The remaining authors declare that the research was conducted in the absence of any commercial or financial relationships that could be construed as a potential conflict of interest.

## Publisher's note

All claims expressed in this article are solely those of the authors and do not necessarily represent those of their affiliated organizations, or those of the publisher, the editors and the reviewers. Any product that may be evaluated in this article, or claim that may be made by its manufacturer, is not guaranteed or endorsed by the publisher.
